# Severe Postoperative Pain in Total Knee Arthroplasty Patients: Risk Factors, Insights and Implications for Pain Management via a Digital Health Approach

**DOI:** 10.3390/jcm12247695

**Published:** 2023-12-15

**Authors:** Julien Lebleu, Andries Pauwels, Hervé Poilvache, Philippe Anract, Anissa Belbachir

**Affiliations:** 1 moveUP, Cantersteen 47, 1000 Brussels, Belgium; 2 Orthopedic Surgery Department, CHIREC, 1420 Braine-l’Alleud, Belgium; 3 Service de Chirurgie Orthopédique, Hopital Cochin, Université Paris Cité, 75014 Paris, France; 4 Service d’Anesthésie, Réanimation et Médecine Périopératoire, Hopital Cochin, Université Paris Cité, 75014 Paris, France

**Keywords:** knee surgery, chronic pain, mhealth, analgesia, pain trajectory, pain management, prehabilitation

## Abstract

Up to 25% of patients undergoing knee arthroplasty report chronic pain postoperatively. Early identification of high-risk individuals can enhance pain management strategies. This retrospective analysis investigates the incidence of severe postoperative pain and its associated risk factors among 740 patients who underwent total knee arthroplasty. Utilizing a digital application, patients provided comprehensive data encompassing pre- and postoperative pain levels, analgesic usage, and completed a chronic pain risk assessment. Participants were categorized into two distinct groups based on their pain status at three months post-op: Group D+ (14%), characterized by pain scores exceeding 40/100 and/or the utilization of level 2 or 3 analgesics, and Group D− (86%), who did not meet these criteria. An analysis of pain trajectories within these groups revealed a non-linear progression, with specific patterns emerging amongst those predisposed to chronic pain. Notably, patients with a trajectory towards chronic pain exhibited a plateau in pain intensity approximately three weeks post-surgery. Significant preoperative risk factors were identified, including elevated initial pain levels, the presence of comorbidities, pain in other body areas, heightened joint sensitivity and stiffness. This study highlights the utility of digital platforms in enhancing patient care, particularly through the continuous monitoring of pain. Such an approach facilitates the early identification of potential complications and enables timely interventions.

## 1. Introduction

Despite the implementation of multimodal pain management techniques, the postoperative phase following total knee arthroplasty (TKA) frequently results in significant pain for patients. This often leads to adverse patient outcomes, including dissatisfaction, prolonged hospitalization, diminished quality of life, and non-adherence to rehabilitation protocols [1]. Additionally, the extended use of analgesics can precipitate adverse side effects [2], contributing to the development of chronic pain symptoms in 10 to 30% of cases [3,4].

The current understanding of the risk factors contributing to chronic pain post-TKA is incomplete, despite extensive research efforts [5,6]. Research has identified several potential risk factors, including local inflammation due to surgery, nerve sensitization, psychological factors such as anxiety and depression, pain catastrophizing, and preoperative opioid use [7,8,9,10]. Accurately predicting which patients will develop chronic pain remains a significant challenge. However, recent studies have highlighted the potential of perioperative pain patterns as a valuable indicator for identifying patients at risk of chronic pain [11,12,13].

The past decade has seen the advent of digital health technologies enabling the continuous collection of perioperative data [14,15,16,17], including pain scores [18].

This development has provided physicians with enhanced insights into patient pain experiences, thereby improving pain management strategies. By integrating these technologies with the knowledge of identified risk factors, there is potential for a more profound understanding and prediction of chronic pain post-TKA [19,20].

This study aims to: (1) characterize the structure of acute pain trajectories during the postoperative period following TKA using a digital platform, (2) investigate risk factors potentially leading to chronic pain, and (3) examine the differences in recovery patterns among patients who develop chronic pain.

## 2. Materials and Methods

### 2.1. Study Design and Data Source

We conducted a retrospective observational study using anonymized depersonalized data from the database of moveUP digital therapies (moveUP solution, Brussels, Belgium). The database comprises data from patients who underwent hip and knee arthroplasty across Belgium, France, and the Netherlands. A cohort of 740 patients who underwent elective total knee arthroplasty was selected, covering the period from November 2017 to June 2023. Patients were included in the study if they used the digital application for at least 70 days after surgery and completed their patient reported outcome measures at 3 months postop. Prior to the inclusion, each patient provided written informed consent for the scientific use of their anonymized data. Regulatory guidelines were followed with no involvement of institutional review board (IRB) approval as this study used anonymized patient-level data.

### 2.2. Data Collection System

All data collection was facilitated via the moveUP^®^ application (moveUP^®^, Brussels, Belgium), which is registered as a medical device. This application operates on a smart virtual platform designed for digital monitoring, utilizing both objective and subjective patient data. The platform consists of two main components: a patient-facing mobile application and a web-based dashboard utilized by the care provider.

**Figure 1:**
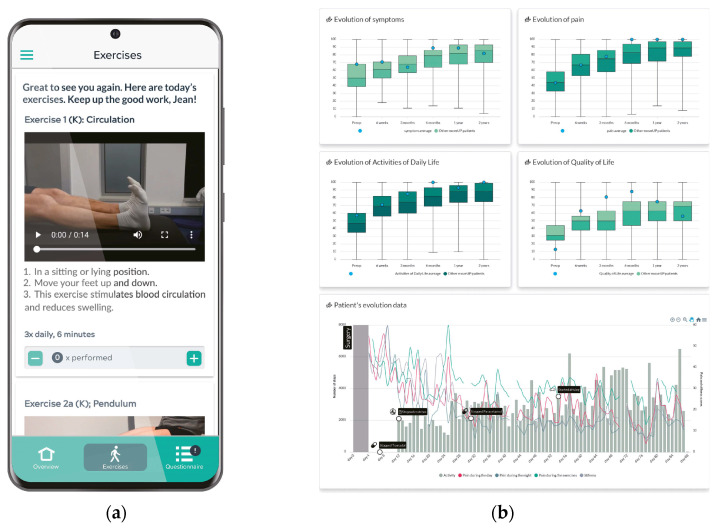
Patient application (**a**) and example of an evolution report showing patient reported outcomes scores against a population and the evolution of daily data (**b**).

In concurrence with data collection, the application provided patients with timely educational materials regarding their postoperative recovery. These informational resources were developed and validated in collaboration with hospital care teams [14], ensuring the delivery of credible and accurate guidance to facilitate patient recovery (Figure 1).

### 2.3. Outcomes

The patients used the moveUP app to collect pre- and postoperative pain data, chronic pain risk factors, analgesic usage and physical activity (using a commercial activity tracker (Garmin Vivofit 4) worn 24/7 by the patients.). Pain and swelling were assessed daily using a visual analog scale (VAS: 0–100 scale, higher score indicating worse pain) [21]. Pain medication type and frequency was also assessed daily (level 1, 2 and 3 painkillers).

Potential chronic pain risk factors were collected pre-operatively: age, gender, body mass index (BMI), type and number of comorbidities, patient reported sensitivity, patient reported stiffness, preoperative pain intensity at rest, preoperative pain intensity at night, and presence of pain elsewhere at multiple sites (Table 1). Patient-reported outcomes such as Knee Osteoarthritis Outcome score (KOOS), and EuroQol 5-Dimension (EQ5D) were measured before surgery and 6 weeks, 3 months, 6 months, 1 and 2 years after surgery through the app.

### 2.4. Chronic Pain

The International Association for the Study of Pain (IASP) defines chronic post-surgical pain as pain that persists beyond the healing process, i.e., at least three months after surgery [22]. Patients were divided into two groups: those exhibiting pain scores greater than 40/100 and/or consuming level 2 or 3 analgesics were categorized as D+, and those who did not fulfill these criteria at three months post-surgery were categorized as D− [23].

### 2.5. Statistical Analysis

Descriptive statistics were used to describe the data with median (interquartile range) for continuous variables, and number (percentage) for categorical variables. The Chi-square test was applied to assess proportional differences between groups. The Mann–Whitney U test was utilized to compare the parameters between the groups, with an alpha error threshold set at 0.05.

## 3. Results

Demographic characteristics of the study participants are delineated in Table 2. Within the cohort, the non-chronic pain group (D−) constituted 632 patients (85%), whereas the chronic pain group (D+) comprised 108 patients (15%). At the three-month postoperative interval, 89 patients reported a pain intensity exceeding 40/100, and 19 patients were using level 2 or 3 analgesics. The majority of participants in both cohorts were retired. A higher proportion of females was observed in the chronic pain group (71%) compared to the non-chronic pain group (56%).

**Figure 2:**
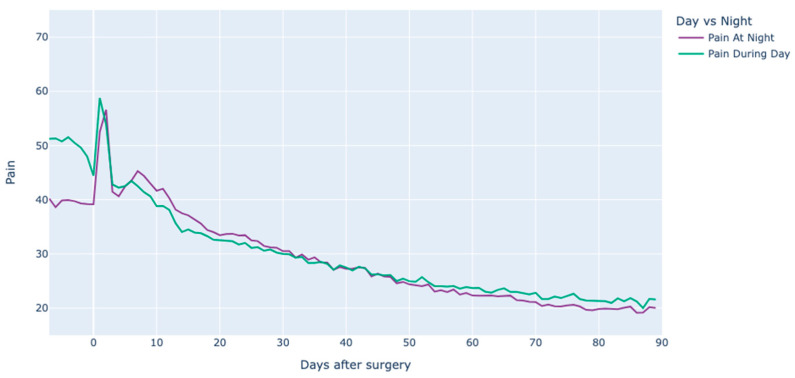
Pain intensity evolution during day and night over the 90 days post-surgery for the whole TKA population.

Preoperative pain intensity was reported to be greater during the daytime as opposed to nighttime (Figure 2). A notable peak in pain was recorded within the initial two days post-surgery, which then subsided by the third day and approached a plateau around day 45.

**Figure 3:**
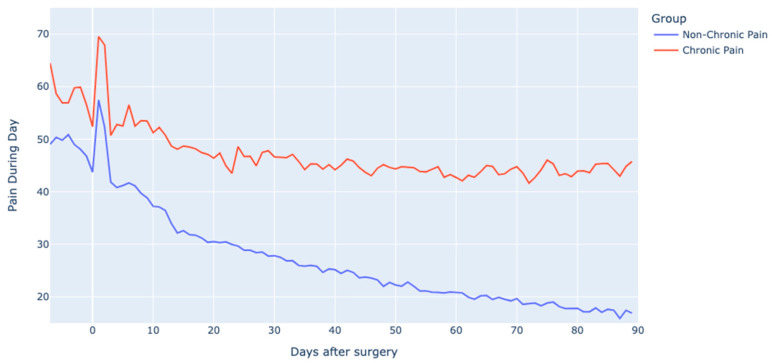
Pain experienced during the day for the chronic and non-chronic pain group. Rel to surgery: relative to surgery.

During the first and second weeks post-surgery, the chronic pain cohort consistently reported elevated pain levels relative to the non-chronic pain cohort, with these disparities proving to be statistically significant. Over time, the divergence in pain trajectories became more pronounced (Figure 3). The pain levels in the chronic pain cohort began to plateau after three weeks, while those in the non-chronic pain cohort continued on a declining trend.

Comparative analysis revealed significant statistical differences across most measured parameters between the chronic pain group (D+) and the non-chronic pain group (D−) as presented in Table 3. Patients in the chronic pain cohort were more likely to have at least one comorbidity, with 79% of them reporting such conditions.

Furthermore, a substantial proportion of the chronic pain group reported experiencing pain in areas other than the surgical site (60% in D+ vs. 42% in D−). This group also demonstrated statistically significant higher levels of sensitivity, stiffness, and pain at night (*p*-value < 0.05). A higher proportion of the chronic pain group experienced moderate and severe pain than the non-chronic pain group [24].

When considering preoperative patient-reported outcome measures, those with chronic pain showed significantly lower scores, particularly in KOOS Pain, KOOS Activities of Daily Life, and KOOS Quality of Life domains (*p*-value < 0.05).

**Figure 4:**
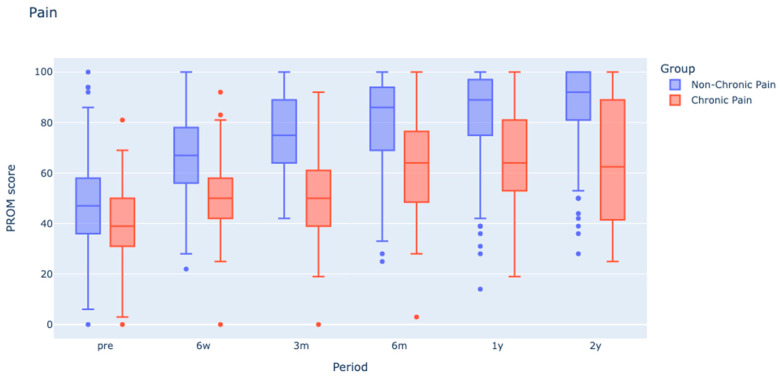
Box plots illustrating the distribution of KOOS pain in chronic and non-chronic pain groups over a 2-year duration. PROM: Patient reported outcome score for KOOS pain.

Postoperatively, the chronic pain group consistently scored lower across all KOOS subscales when compared to their counterparts without chronic pain (*p* < 0.05), except for the KOOS Symptoms domain, which only diverged at 6 months, and one and two years post-surgery (*p* < 0.05), as indicated in Figure 4.

**Figure 5:**
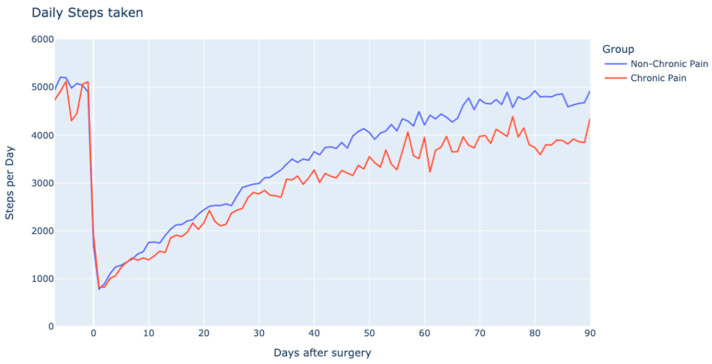
Recovery of physical activity level (number of steps/day) in chronic and non-chronic pain groups. Rel to surgery: relative to surgery.

**Figure 6:**
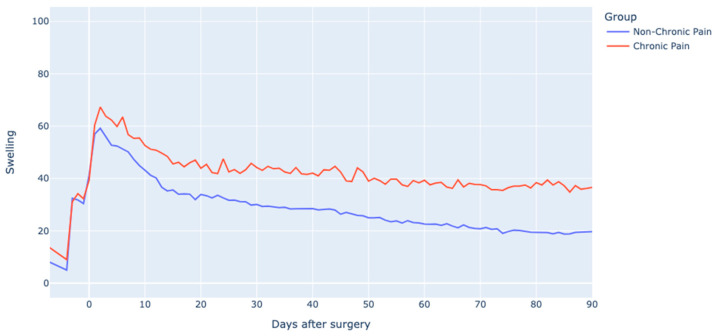
Patient reported swelling (VAS scale 0–100) in chronic and non-chronic pain groups. Rel to surgery: relative to surgery.

Preoperative physical activity levels, approximating 5000 steps/day, were comparable between the groups. Any differences in early postoperative physical activity were minimal, but tended to grow over time (Figure 5). Similarly, patient-reported swelling showed no preoperative variance but escalated as time progressed (Figure 6). The duration of NSAID use differed between the groups; patients with chronic pain used NSAIDs for a median of 51 days (IQR 31–76 days), while those without chronic pain had a shorter duration of 33 days (IQR 21–58 days).

## 4. Discussion

The primary objectives of our investigation were to: (1) characterize the structure of acute pain trajectories post-surgery, (2) identify risk factors potentially leading to chronic pain, and (3) assess recovery patterns in patients with chronic pain post-TKA.

The concept of pain trajectories is a relatively recent development, distinct from the traditional approach of assessing pain at discrete time points [11]. Pain does not follow a linear progression over the 90 days following surgery; instead, it tends to follow a distinct pattern [25]. After surgery, there is an acute pain phase as a reaction to the surgical trauma, followed by an early recovery around day 3 with a gradual reduction in the peak pain. Subsequently, the pain continues to decrease gradually, eventually reaching a plateau that many patients describe as a manageable level of pain. However, it is important to note that not all patients follow this pattern, and some require specific attention. The transition from subacute to chronic pain is of significant research interest. In the Althaus study [11], pain resolution at 5 days in one of the patient groups did not occur despite analgesic treatment. Previous analyses suggest that psycho-social variables such as depression and anxiety may play a role in pain resolution by influencing degree of support [26].

By dividing the population into two groups, we gained a better understanding of the typical pain progression for patients who ultimately experience chronic pain. Notably, these patients deviated from the expected pattern of diminishing pain intensity, instead reaching a plateau in pain levels by the third postoperative week. Furthermore, this unfavorable trajectory is reflected in worse KOOS pain scores at 2 years post-surgery. The total knee arthroplasty (TKA) population is inherently diverse, and this arbitrary binary classification underscores the necessity for personalized, person-centered care.

The link between high preoperative pain intensity and the evolution of chronic pain postoperatively was reinforced, with our chronic pain cohort exhibiting elevated initial pain levels [5,6]. This suggests a heightened nociceptive response or an increased sensitivity of the pain signaling system. Nevertheless, the marginal differences in initial pain between the groups preclude its utility as a standalone prognostic marker. In contrast, the divergence in pain levels at two and three weeks postoperative offers a more definitive prognostic indicator, signaling an optimal period for clinical intervention. Four studies already investigated the association between pain severity during the first two weeks and chronic pain, but there was insufficient evidence to draw firm conclusion [27]. The question remains about why these two groups exhibit such divergent pain resolution trajectories.

The study confirmed that having pain in multiple areas or other health issues increases the risk of chronic pain post-TKA, which aligns with previous research [4,5,6]. Predictive models using simple risk factors show promise in identifying patients at risk for chronic pain [28]. Yet, this study did not capture data on neuropathic pain or psychological factors like pain catastrophizing, depression, and anxiety, which are known to affect pain perception and recovery [29].

We split the TKA patients into groups based on pain at the three-month mark, because the IASP identifies this as when acute pain may become chronic. Our analysis shows it is unnecessary to wait six months to see if a patient’s recovery is off track. Most improvement happens in the first three months post-surgery [30,31], and higher pain scores at three months could indicate worse outcomes at one and two years. Singh et al. observed similar patterns, with distinct pain trajectories at eight weeks predicting six-month outcomes [13]. While most patients recover typically, some have different experiences up to eight years post-surgery [32].

Our chronic pain incidence was 14%, lower than the 30% found in some studies [23]. Ongoing pain tracking could help clinicians spot problems and tailor treatments. Digital tools are already making strides in patient education, reducing post-TKA pain [16]. A stronger digital link between patients and care providers, including automatic alerts for quick response by pain management teams, might further cut chronic pain rates in a cost-effective manner.

Unfortunately, information regarding the type of anesthesia used in the 40 different centers was not available. While the specific anesthesia details may enhance the granularity of our analysis, we contend that the scale and diversity of our dataset allow for valuable insights into broader trends and practices. However, as regional anesthesia techniques, such as epidural anesthesia [33] and fascial plane blocks [34], have been employed to alleviate acute pain after TKA, this may limit the generalizability of our study. Indeed, while these techniques primarily target perioperative pain, there is evidence suggesting that effective pain control during the immediate postoperative period may have implications for the development of chronic pain [33,34,35,36]. The potential mechanisms underlying the observed reduction in chronic pain incidence involve the prevention of central sensitization and modulation of the inflammatory response. Regional anesthesia techniques, by targeting specific nerve pathways, may contribute to a more controlled perioperative pain environment, mitigating the neuroplastic changes associated with chronic pain development [37]. Furthermore, the attenuation of the inflammatory response during the early stages of recovery may have implications for long-term pain outcomes [38].

**Figure 7:**
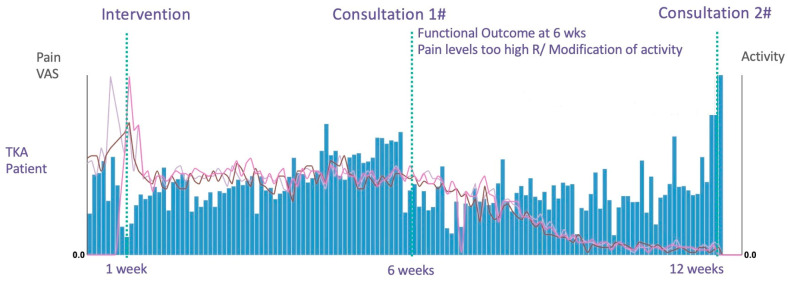
Patient case. Blue bars represent the level of physical activity. The lines represent the pain and the swelling.

Pain is linked to swelling in the acute phase, which is influenced by how active patients are [39]. To manage this, patients received custom advice from physical therapists. They used standard inflammation control like ice, activity management, and NSAIDs. These approaches may affect pain over time. Despite measures to control inflammation, the chronic pain group still showed more swelling by a score of 20/100 at three months post-surgery compared to their counterparts. The impact of these interventions is more visible on an individual level, as illustrated by a case where a patient’s early resumption of physical activity led to stagnant pain and swelling levels. Following the advice to significantly reduce physical activity after the first consultation, the patient experienced a decrease in pain after a few weeks. While instructive, more research is needed to clarify the influence of activity pacing and NSAID use on postoperative swelling and pain (Figure 7).

The study has several limitations. Its retrospective design can show associations but not causality. Prospective studies are necessary to understand the cause-anD−effect relationship between risk factors and chronic pain. The selection of participants may also introduce bias, as it relies on patients consistently using a mobile app for data collection, potentially excluding those who recovered quickly or did not use the app. Additionally, while the study points to pre-surgery risk factors for chronic pain, it may not account for all variables that could affect pain outcomes. The study also did not differentiate between the specific causes of chronic pain, such as extra-articular or intra-articular issues [40], which might influence results and introduce confounders. Further research is required to parse out these distinctions for a clearer understanding of chronic pain development after TKA.

## 5. Conclusions

In this research, we conducted a detailed examination of acute pain trajectories post-total knee arthroplasty (TKA) and investigated the risk factors for chronic pain. Our results bring to light the complex nature of postoperative pain, stressing the critical role of early detection and individualized management strategies in optimizing outcomes for TKA patients.

The analysis indicated that pain after TKA does not follow a simple, linear decline, but rather presents in various patterns, particularly a plateauing of pain levels after the third week in patients who develop chronic pain. This suggests the necessity for adaptive care plans that are responsive to the unique pain experiences of each patient.

Identified preoperative risk factors for chronic pain included not only high pain levels before surgery, but also the presence of multiple comorbidities, pain in other body areas, increased sensitivity, joint stiffness, and nocturnal pain. These findings are instrumental in forming predictive models that can be used to anticipate chronic pain and customize early interventions.

The study highlights the efficacy of continuous pain monitoring with digital platforms in enhancing patient care. By establishing a digital interface between patients and healthcare providers, there is an opportunity for more immediate detection of complications and the provision of prompt care, which may lead to a decrease in the occurrence of chronic pain.

To conclude, this study advances our understanding of the postoperative pain landscape and the elements that influence chronic pain after TKA. Emphasizing personalized and interdisciplinary approaches, and incorporating digital healthcare tools, this research points towards improved patient outcomes and the elevation of care standards in the field of orthopedic surgery. The insights gained herein pave the way for future research to refine clinical approaches in managing post-TKA pain.

## Figures and Tables

**Table 1:** Questions asked through the application.

Questions	Answer Options
Indicate on the slider the stiffness you experienced in your joint?	0–100 slider—none—very stiff
What is the intensity of your pain?	0–100 slider—no pain—intolerable pain
Does your affected joint feel swollen? (no swelling—very swollen)	0–100 slider—no swelling—very swollen
In the last 7 days, how bad was you pain at rest?	0–100 slider—no pain—worst pain imaginable
In the last 7 days, how bad was your pain at night?	0–100 slider—no pain—worst pain imaginable
On a scale from 0 to 10, how severe is the sensitivity in/around the joint?	0–10 choice—normal- not sensitive at all, extremely sensitive
Do you experience complaints to other areas surrounding your index joint?	Yes—No
Did you take any medication today?	Yes—No
Add the medication you took today below (painkillers, anti-inflammatory, anti-coagulants, etc.)	Multiple choice + free entry

**Table 2:** Demographics comparison between chronic and non-chronic pain groups.

Characteristic	Chronic Pain (D+)	Non-Chronic (D−)	*p*-Value
N (%)	108 (14.6%)	632 (85.4%)	/
Age (years), median (IQR)	61.0 (56.0–70.0)	63.0 (57.0–69.0)	0.989
BMI, median (IQR)	29.0 (26.1–32.6)	29.0 (26.1–32.7)	0.711
Gender, N (%)			0.007
Female	77 (71)	352 (56)	
Male	31 (29)	273 (43)	
Unknown	0	7 (1)	
Employment status, N (%)			0.034
Employed	1 (1)	43 (8)	
Unemployed	88 (98)	497 (92)	
Educational background, N (%)			0.253
Primary school	11 (12)	48 (9)	
Secondary school	47 (52)	260 (48)	
Bachelor degree	20 (22)	139 (26)	
Master degree	9 (10)	88 (16)	
I prefer not to answer this question	2 (2)	4 (1)	

N: number of patients, IQR: interquartile range.

**Table 3:** Risk factors comparison between chronic and non-chronic pain groups.

Characteristic	Chronic Pain (D+)	Non-Chronic (D−)	*p*-Value
Number of comorbidities, %			<0.001
0	21	53	
1	57	31	
>2	22	16	
**Pre-operative parameters**			
Number of patients with pain elsewhere than surgical site, %	60	42	
Percentage of patients in each pain category, %			<0.001
No pain—VAS 0–4	0	1	
Mild pain—VAS 5–44	17	42	
Moderate pain- VAS 45–74	70	55	
Severe pain—VAS 75–100	13	2	
Sensitivity (0/10) (median, IQR)	8.0 (7.0–8.0)	7.0 (6.0–8.0)	0.002
Stiffness (0/100) (median, IQR)	59.3 (49.3–73.0)	46.2 (31.6–59.8)	<0.001
Pain at night (0/100) (median, IQR)	52.8 (41.0–63.4)	36.7 (22.7–51.3)	<0.001
Swelling (0/100) (median, IQR)	29.8 (12.9–53.6)	25.0 (11.4–50.7)	0.40
Physical activity (number of steps) (median, IQR)	3637 (2545–5490)	3916 (2262–5931)	0.75
**Peri-operative parameters**	Chronic pain (D+)	Non-Chronic (D−)	
Pain first week (0/100) (median, IQR)	52.6 (43.4–62.7)	37.6 (27.7–47.4)	<0.001
Pain second week (0/100) (median, IQR)	48.1 (41.2–56.8)	31.2 (22.6–41.4)	<0.001
**Patient reported outcomes**	Chronic pain (D+)	Non-Chronic (D−)	
EQ5D (VAS score) (median, IQR)	54.0 (38.0–72.0)	62.0 (43.0–76.0)	0.009
KOOS Pain (median, IQR)	39.0 (31.0–50.0)	47.0 (36.0–58.0)	<0.001
KOOS Symptoms (median, IQR)	50.0 (39.0–64.0)	54.0 (43.0–68.0)	0.47
KOOS Activities of Daily Life (median, IQR)	40.5 (31.8–51.5)	50.0 (39.5–63.0)	<0.001
KOOS Quality of Life (median, IQR)	19.0 (6.0–31.0)	25.0 (19.0–38.0)	<0.001

N: number of patients, IQR: interquartile range, VAS: visual analogic scale.

## Data Availability

The data presented in this study are available on request from the corresponding author. The data are not publicly available due to privacy reasons.
